# Optical Camera Communications in Healthcare: A Wearable LED Transmitter Evaluation during Indoor Physical Exercise †

**DOI:** 10.3390/s24092766

**Published:** 2024-04-26

**Authors:** Eleni Niarchou, Vicente Matus, Jose Rabadan, Victor Guerra, Rafael Perez-Jimenez

**Affiliations:** 1 Institute for Technological Development and Innovation in Communications (IDeTIC), University of Las Palmas de Gran Canaria, 35001 Las Palmas de Gran Canaria, Spain; vicente.matus@ulpgc.es (V.M.); jose.rabadan@ulpgc.es (J.R.); rafael.perez@ulpgc.es (R.P.-J.); 2 Pi-Lighting, 1950 Sion, Switzerland; victor.guerra@pi-lighting.com

**Keywords:** optical camera communications (OCC), wearable devices, image processing, exercise analysis

## Abstract

This paper presents an experimental evaluation of a wearable light-emitting diode (LED) transmitter in an optical camera communications (OCC) system. The evaluation is conducted under conditions of controlled user movement during indoor physical exercise, encompassing both mild and intense exercise scenarios. We introduce an image processing algorithm designed to identify a template signal transmitted by the LED and detected within the image. To enhance this process, we utilize the dynamics of controlled exercise-induced motion to limit the tracking process to a smaller region within the image. We demonstrate the feasibility of detecting the transmitting source within the frames, and thus limit the tracking process to a smaller region within the image, achieving an reduction of 87.3% for mild exercise and 79.0% for intense exercise.

## 1. Introduction

Optical wireless communications (OWC) stand as a significant area of exploration in mobile communication, offering advantages such as cost effectiveness, high-speed capabilities, and reliable data transmission [1]. Already acknowledged as a complementary or sometimes viable alternative to radio-frequency (RF) technology, OWC includes promising technologies such as optical camera communications (OCC). OCC employs a light-emitting diode (LED) as the transmitter ($T_{x}$), an image sensor (IS) (i.e., camera) as the receiver ($R_{x}$), and light as as the signal communication carrier. This approach boasts several valuable attributes, including low cost, high security, low power consumption, and enhanced reliability. Importantly, it is devoid of electromagnetic interference, ensuring complete safety for human health [2]. The extensive deployment of smart devices, not only smartphones that have built-in complementary metal oxide semiconductor (CMOS) cameras and are all interconnected within the Internet, has paved the way for innovative applications of OCC and serves as a cornerstone for the development of OWC-based Internet of Things, termed optical IoT (OIoT) [3]. These applications include indoor positioning systems [4], underwater [5], localization [6], and healthcare applications [2].

Smart devices, encompassing smartphones, smartwatches, and smart clothing, are recognized as products that seamlessly incorporate wearable technologies to distinguish human activities [7]. Wearable devices, designed to be lightweight and compact, offer user convenience and integrate seamlessly into clothing or accessories or directly attached to the body (like glucose sensor patches) without disrupting daily activities. Equipped with sensors, processors, and communication capabilities, these devices aim to provide specific functionalities, such as monitoring health and fitness metrics [8]. Wearable health-monitoring sensors have become ubiquitous in our daily lives [9,10], playing a crucial role in healthcare systems for real-time and continuous patient health monitoring [10]. They also serve as a cornerstone for the IoT [11]. Sensors measure parameters before the OCC system collects the data, forwarding them to the camera through integrated light-emitting diodes. With the emergence of 6G, the integration of wearables in healthcare is poised to expand, signaling an era of intelligent healthcare [12] characterized by enhanced sensing, processing, and communication capabilities.

To date, only a limited body of research has explored the integration of wearable sensors with LEDs as transmitters. For instance, in [13], medical sensors and infrared LEDs collaborate to transmit medical data for patient monitoring. Similarly, ref. [14] employs this combination for indoor health monitoring, taking into account patient mobility. Additionally, ref. [15] introduces an all-optical bidirectional wireless communication system that evaluates sensor mobility, variations in orientation, and placement on the body. Furthermore, ref. [16] investigates the performance of optical code-division multiple access in asynchronous mode, considering the impact of mobility and random transmitter orientations. Moreover, [17] explores optoelectric sensors monitoring cardiovascular vital signs.

The use of OWC technologies in healthcare tourism has been extensively studied in [18], including the use of this technology for monitoring elder or impaired people with special needs. The use of wearable devices, jointly with location techniques [19], allows detecting whether the user is immobile for long periods, has suffered a fall or a sudden change in vital signs, or is simply leaving a predefined safety zone, in which they can remain without requiring constant attention from their caregiver. For this cases IR emitters can be considered instead of the visible ones, to preserve user privacy in general-purpose environments such as hotels, without loss of generality in this proposal as near-IR can be detected by regular CMOS-Silicon based cameras [20].

In the field of OCC, few works have been done considering wearables as transmitters. In our previous research, we showcased a wearable LED array [21] and a fiber attached on T-shirt [22] as distributed transmitters. Recently, there has been notable development in various medical applications that focus on using wearable sensors to measure individuals’ health conditions. For instance, in [2], a system has been implemented for real-time remote monitoring of a patient’s heart rate and oxygen saturation data. Similarly, in [23], a system facilitates the transmission of multiple clinical data types, including electrocardiogram, photoplethysmogram, and respiration signals in a home-based rehabilitation system. In addition, OCC has demonstrated its adaptability by being combined with other technologies, giving rise to hybrid systems that leverage the strengths of each technology, thereby enhancing their robustness [24]. Specifically, in [25], OCC is integrated with Bluetooth Low Energy (BLE) to enable efficient, remote, and real-time transmission of a patient’s electrocardiogram signal to a monitor. A similar combination is explored in [26] for real-time health monitoring, where data from body sensors is transmitted to a central gateway. In cases where node movement in OCC can disrupt the connection, BLE steps in to ensure continuous communication.

Analysis of human exercise routine data can provide valuable insights. For instance, in [27], a smart exercise bike was developed specifically for rehabilitation from Parkinson’s disease. Another example is found in [28], where a camera-based monitoring system offers indications for cardiovascular health and optimizes training protocols. Additionally, ref. [29] introduces a video-based heart rate detection system to monitor people’s heart rates during exercise. Moreover, ref. [30] introduces a monitoring system for elderly people is introduced, capable of autonomously identifying significant deviations in their presence pattern. Furthermore, in [31] the proposed system determines body posture and identifies the physical condition and health of the body. Moreover, ref. [32] presents a machine learning-based analysis of the typing pattern analysis detects depressive disorder. Similarly, ref. [33] explores the analysis of keyboard interactions recorded on an individual’s smartphone can offer valuable insights into the clinical status of multiple sclerosis. Lastly, ref. [34] investigates keystroke dynamics for the early detection of loneliness and the development of targeted interventions.

In this study, we conduct an experimental evaluation of an OCC system utilizing a wearable LED transmitter. The evaluation assumes controlled user movement during physical exercise in an indoor setting. The wearable LEDs are modulated in intensity to transmit binary data, imperceptible to the human eye but detectable by a smartphone camera operating at a specific frequency. The camera tracks the user’s movements and captures the transmitted data.

Our focus is on addressing challenges related to transmitter detection and tracking [35]. To achieve this, we propose employing a template signal transmitted by the LED, denoted as $T_{x}$, and detected in the image through a correlation process. This information will serve a dual purpose aligned with the Integrated Sensing And Communication (ISAC) paradigm. The main hypothesis is that the user’s position (i.e., $T_{x}$’s detection) within the frame correlates with factors such as exercise intensity, age, gender, etc. This correlation may be even more profound, suggesting individual differences and the potential to detect chronic conditions or even early signs of injuries. Further exploration of this hypothesis will be conducted in subsequent phases of the research, utilizing the acquired data. To simplify the $T_{x}$’s detection process within the frame, we leverage the characteristics of controlled exercise-induced movement, confining the tracking process to a smaller area within the image.

Our envisioned system is designed to monitor the activities of individuals who are either in good health or those who face health problems. This monitoring can take place in various environments such as homes, gyms, ambulances, hospitals, and intensive care units [12,36]. Consequently, it has the potential to aid in rehabilitation, sports training, elderly care [37], early detection of musculoskeletal or cognitive diseases, and evaluations of falls and balance. The main innovation of this study revolves around employing widely accessible and commercially available wearable devices, including LEDs, and integrating them with smartphones for communication purposes.

The structure of the paper is outlined as follows. Section 2 describes the system designed, with the equipment employed in both the transmitting and receiving nodes and the experimental setup. Section 3 examines the methodology, including the image processing and the analysis of the user’s exercise. Section 4 discusses the experimental results obtained. Ultimately, Section 5 presents the conclusions drawn from this work.

## 2. System Design

**Figure 1:**
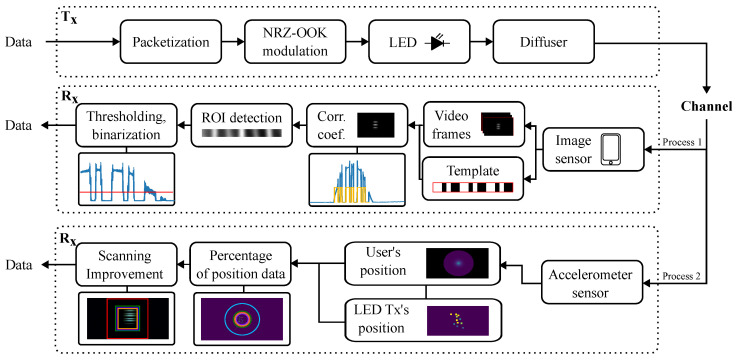
Block diagram of the transmitting and receiving node.

In this section, we provide an overview of the equipment utilized in both transmitting and receiving nodes of the proposed system. Additionally, we provide a detailed description of the experimental setup. The block diagram of the proposed OCC link is shown in Figure 1.

The system utilized for the envisioned experiment included digital signal processing hardware and optical front-ends. The $T_{x}$ consisted of a standard LED device linked to the digital output of a micro-controller unit (MCU) (Seeeduino Xiao [38]). The devise is comprised of 30 white LEDs, rechargeable batteries of 5 V, and a diffuser. The LED’s transmitted illuminance at 0 cm measured with testo 545 lux meter, is 17,443 lux, while the received illuminance at 25 cm is 105 lux.

The proposed OCC system utilizes the non-return-to-zero on-off keying (NRZ-OOK) modulation technique for transmitting data wirelessly across a free-space channel. Employing the digital switching outputs of the micro-controller unit (MCU), the system facilitates NRZ-OOK modulation [39]. The $T_{x}$ device is modified accordingly in order to drive the LEDs with a transistor powered directly from the battery terminals. The MCU generates a 6-bit data packet [110100] at a rate of 0.4 ms per bit, corresponding to a modulation frequency of 2.5 kHz per bit. This data packet is transformed into a voltage signal, directly driving the LEDs. To overcome the MCU’s maximum current limit, a transistor is connected to the power source for LED driving. To enhance link performance, a repeat-packet strategy is implemented.

On the other hand, the $R_{x}$ was a smartphone [40] camera which captures video in rolling shutter (RS) mode. The RS-based cameras can capture the image row-by-row of pixels, which means that different lines of the image array are exposed at various times to read the light intensity through the sensor enabling multiple states of LEDs (ON and OFF) can be obtained in a single frame [41]. The smartphone camera captures video from a distance of 20–30 cm. The smartphone camera captures a 30 fps frame-rate video, with exposure time of 83 μs, and ISO 125 [42], using resolution (7680 × 4320 px). The exposure time is the time the camera is exposed to light and the ISO number refers to to the amount of light the camera lets on the sensor. The most relevant parameters of the proposed system are summarized in Table 1. It is important to note that all measurements were performed under indoor ambient lighting conditions.

**Figure 2:**
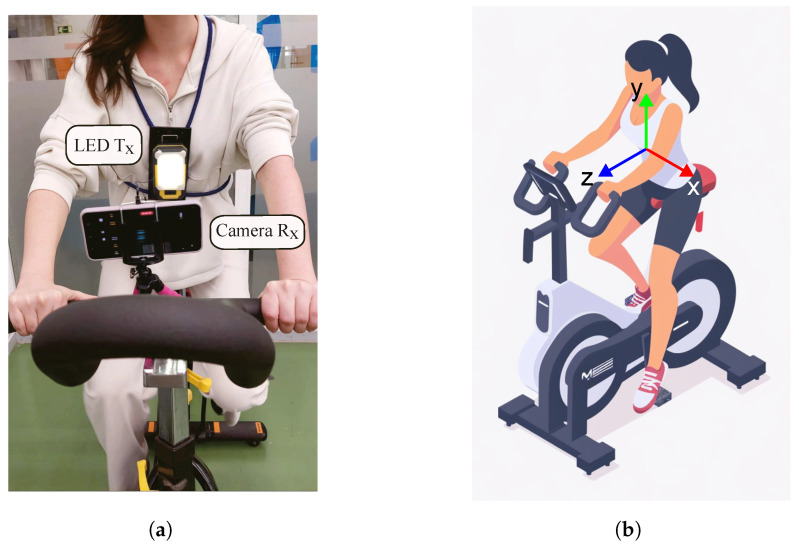
Experimental setup with the wearable transmitter device and the smartphone camera receiver. (**a**) The user engages in physical exercise on a stationary bicycle. (**b**) 3D reference dimensions of the system.

For the evaluation of the OCC system, the person wearing the $T_{x}$ participated in a controlled exercise session on a stationary bicycle. The experimental setup featuring the wearable $T_{x}$ and the $R_{x}$ attached on the bicycle, is illustrated in Figure 2.

The recorded video undergoes offline processing, with the main objective being the detection and tracking of the $T_{x}$. To achieve this, we use a template signal emitted by the LED, which is then identified within the image through a correlation procedure, as shown in Process 1 in Figure 1. To simplify Process 1, we leverage the characteristics of controlled exercise-induced movement in Process 2, thereby limiting the tracking process to a smaller area within the image. Both processes will explained in the next section.

For the exercise scenario we replaced the LED $T_{x}$, with a smartphone, and employed an accelerometer application to measure acceleration data during the exercise. Two types of measurements were conducted, involving the user performing mild and intense exercise routines. Our reference system is depicted in Figure 2b.

## 3. Methodology

In this section, we elaborate on the methodology employed for this experimental setup. Firstly, we analyze the image processing, along with demodulation and data acquisition. Following that, we provide a detailed analysis of exercise-related data within the context of our experimental setup.

### 3.1. Image Processing

In the image processing procedure in Figure 1 (Process 1), the video is first segmented into frames, and a single frame is chosen while a template is generated. This template comprises three consecutive packets, each containing a sequence of [110100] bits. Due to the RS effect, the data rate of the OCC using a CMOS camera can be significantly increased [43].

**Figure 3:**
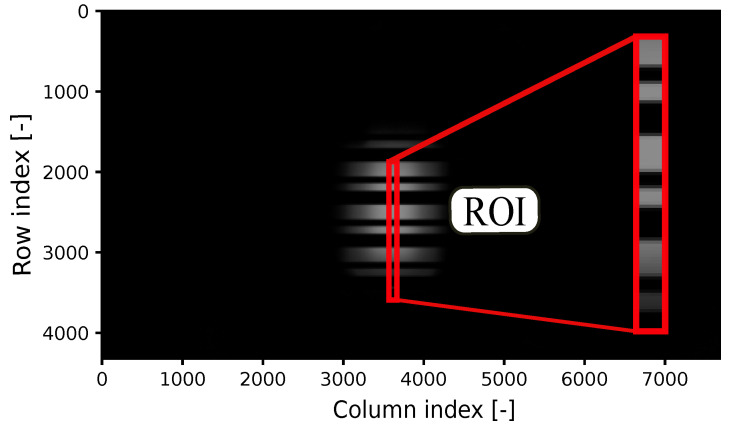
Frame showing values obtained from the correlation coefficient between a random frame and the template. The region of interest (ROI) is highlighted in red.

**Figure 4:**
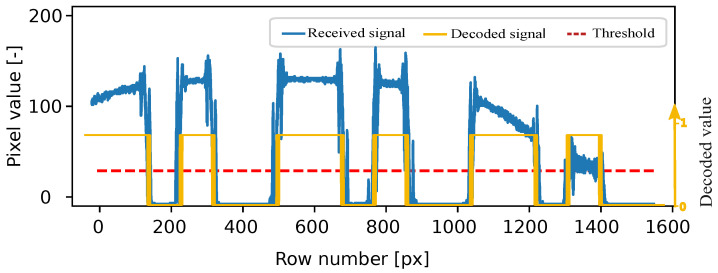
Received grayscale signal, decoded signal, and threshold.

Afterward, the image frames are converted to grayscale, facilitating the extraction of the pixel intensity profile. The correlation process involves sliding the template image over the frame, akin to a 2D convolution, to pinpoint the 2D position of the signal captured from the transmitting source. The blue lines within the inset of the $R_{x}$ section of the block diagram represent the average row value, while the orange line depicts the template signal, and the red line illustrates the binarization threshold. In Figure 3, the region of interest (ROI) in the frame, where the correlation attains the maximum value, is highlighted. This process is carried out iteratively for all frames. The identified ROI is then used for data decoding. Through the application of thresholding and binarization to the acquired data, the received signal is effectively decoded, as shown in Figure 4.

### 3.2. Exercise Analysis

For the exercise scenario mentioned above, our aim is to gain insight into the dynamics of the exercise and capture the exercise routine. To achieve this, we make two assumptions. Firstly, it is assumed that the individual’s average position $\langle \vec{r}\left(t\right)\rangle$ during the workout corresponds to the initial position ${\vec{r}}_{0}$ as shown in Equation (1), simplifying the analysis by considering the average position as the starting point.

(1) $$\langle \vec{r}\left(t\right)\rangle ={\vec{r}}_{0}$$

Secondly, the analysis acknowledges the presence of inertial measurement unit (IMU) error and accounts for cumulative errors in Equation (2) that may cause a drift in position data over time. Despite controlled movement, factors such as sensor inaccuracies can introduce cumulative errors, which are considered in the analysis.

(2) $$\langle \vec{r}\left(t\right)\rangle ∼N\left({\vec{r}}_{0}+{\vec{\mu }}_{N}\cdot t,{\vec{\sigma }}_{N}\cdot t\right)$$

where ${\vec{\mu }}_{N}$ and ${\vec{\sigma }}_{N}$ are the vectors derived from the IMU’s uncertainties with respect to drift and noise, respectively. Analyzing the acceleration data $\vec{a}\left(t\right)$, we obtain the position of the user in Equation (3) by double integrating the acceleration, where ${\vec{v}}_{0}$ is the initial velocity (assumed in $\vec{0}$ at the beginning of the routine).

(3) $$\vec{r}\left(t\right)=\int_{0}^{t}\int_{0}^{t}\vec{a}\left(t\right)dtdT=\int_{0}^{t}\left(\vec{v}\left(t\right)-{\vec{v}}_{0}\right)\;dt$$

The velocity $\vec{v}\left(t\right)$ at discrete time intervals jΔt in Equation (4) is a sum of acceleration $a_{x}\left(i\right)$ and $a_{y}\left(i\right)$ with the time interval Δt along the *x* and *y* directions, respectively. We focus on the XY plane since it is the camera’s plane and no additional information is needed a paior for extracting information in the sensing pathway of the ISAC-enabled reception routines.

(4) $$\vec{v}\left(j\Delta t\right)=\Delta t\sum_{i=0}^{j}a_{x}\left(i\Delta t\right)\cdot {\vec{n}}_{x}+a_{y}\left(i\Delta t\right)\cdot {\vec{n}}_{y}$$

Similarly, the position $\vec{r}$ at discrete time intervals kΔt in Equation (5) is a sum of velocity $v_{x}\left(j\right)$ and $v_{y}\left(j\right)$ with the time interval Δt along the *x* and *y* directions, respectively.

(5) $$\vec{r}\left(k\Delta t\right)=\Delta t\sum_{j=0}^{k}v_{x}\left(j\Delta t\right)\cdot {\vec{n}}_{x}+v_{y}\left(j\Delta t\right)\cdot {\vec{n}}_{y}$$

**Figure 5:**
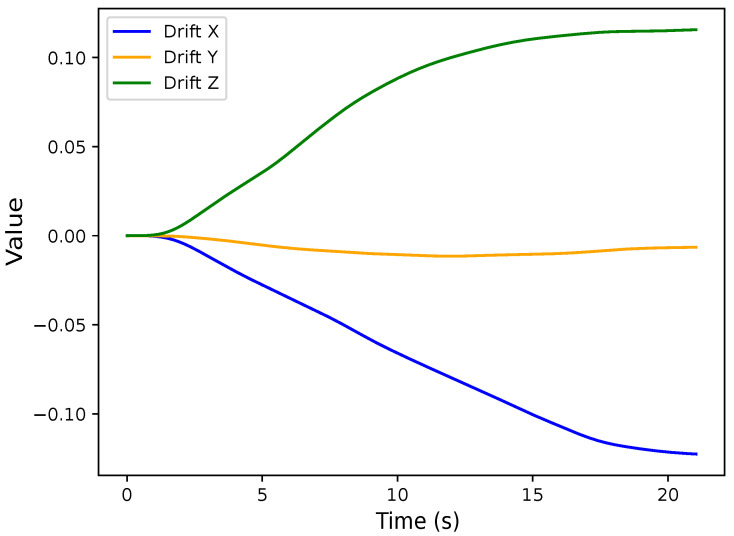
Drift in position data in 3D direction in axis x, y and z.

Using Equation (5), the drift behavior of the IMU was analyzed after capturing 25 s of calibrated acceleration data (removing gravity). This behavior can be observed in Figure 5, suggesting that any analysis should be carried out within a sliding window. In addition, the duration of that window should be lower than a few seconds to avoid any disruption due to cumulative errors. 

The expected value of the position can be calculated as shown in Equation (6), introducing Equation (5) into Equation (4)

(6) $$E\left[\vec{r}\left(k\Delta t\right)\right]=\Delta t^{2}\sum_{j=0}^{k}\sum_{i=0}^{j}E\left[a_{x}\left(i\Delta t\right)\right]\cdot {\vec{n}}_{x}+E\left[a_{y}\left(i\Delta t\right)\right]\cdot {\vec{n}}_{y}$$

Using a moving average of the window size *M*, it yields Equation (7).

(7) $$E_{M}\left[\vec{r}\left(k\Delta t\right)\right]=\frac{\Delta t^{2}}{M}\sum_{l=k-(M-1)}^{k}\sum_{j=0}^{l}\sum_{i=0}^{j}\vec{a}\left(i\right)$$

**Figure 6:**
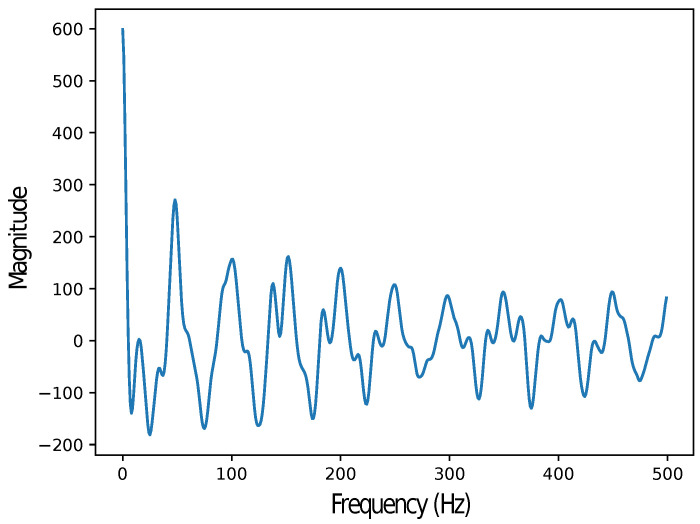
Frequency-domain analysis of the deviation in the XY plane. Data obtained from the IMU.

Some additional assumptions have been made in order to simplify the process. Firstly, reverse to the mean is considered to happen within a given window. Thereby, the averaged position inside that sliding window will be conserved during all the process. This statement holds statistically given the nature of the experimental situation (static cycling). In addition, it has been assumed that it is possible to define the size of the sliding window based on a frequency-domain analysis of the deviation with respect to the average. This analysis, depicted in Figure 6 and mathematically described in Equation (8), suggested that most of the energy is concentrated in the first 47 Hz of the spectrum. This leads to a sliding window length *M* of 47.

(8) $$R_{z}\left(\tau \right)=F^{-1}(F\left(\left(a_{x}\left(t\right)\right)\cdot conj\left(F\left(a_{x}\left(t\right)\right)\right)\right)$$

Following the above calculations and the Process 2 in Figure 1, we determined the frequency of the user’s position in pixels within one frame for both mild and intense exercise scenarios. The results are presented in the following section.

## 4. Results

In this section, we provide a summary of the outcomes derived from applying the image processing algorithm to the video frames obtained, as well as from the analysis of the user’s exercise, during the previously described experiment.

**Figure 7:**
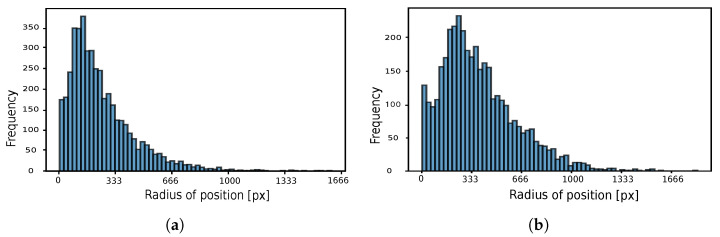
Histograms representing the frequency of a user’s position in pixels within a single frame. Each bin corresponding to the frequency of one position. (**a**) Mild exercise. (**b**) Intense exercise.

**Figure 8:**
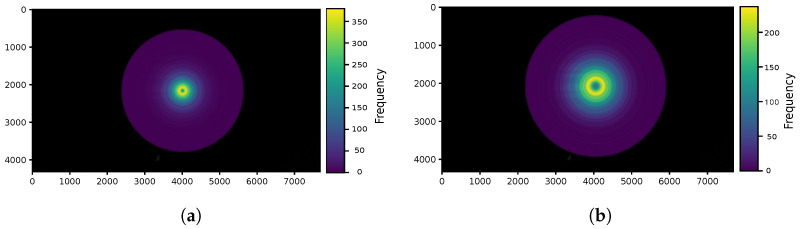
Rings representing the distribution of user’s position within a single frame. Each circle’s radius corresponding to the frequency of the user’s position. (**a**) Mild exercise. (**b**) Intense exercise.

The frequency of the user’s position in pixels within one frame for both mild and intense exercise scenarios is illustrated in Figure 7a and Figure 7b, respectively. Consequently, the user’s position in pixels within one frame can be depicted as the circle’s radius in Figure 8a for mild exercise and in Figure 8b for intense exercise.

**Figure 9:**
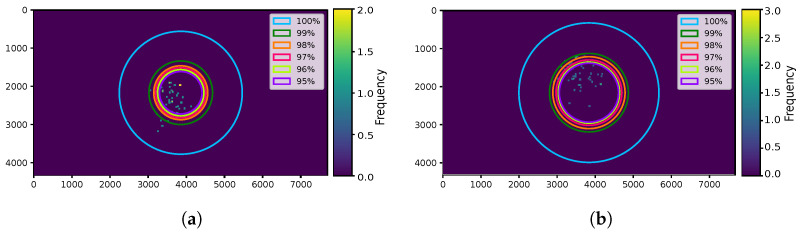
Dots representing the distribution of the LED transmitter’s center within a single frame. Each dot corresponding to the frequency of the LED transmitter’s center. The circles representing the percentage of the position data. (**a**) Mild exercise. (**b**) Intense exercise.

Then, these data, combined with the data obtained from the LED $T_{x}$, provide information about the percentage of the position data we can consider. Considering only the center of the LED $T_{x}$ from previous measurements, we determined the frequency of the center of the LED $T_{x}$ within one frame, represented by the dots in Figure 9a for mild exercise and in Figure 9b for intense exercise. In the same figures, the circles represent the percentage of position data (obtained from the accelerometer), spanning from 100% down to 95%.

From the image processing on the video frames captured with the LED $T_{x}$, we successfully identify the ROI and decode the received signal in all frames, despite the user’s movement within the frame.

By combining these data with the data obtained from the accelerometer, we aim to improve the process of ROI identification by reducing the scanning area in the frame. All the relevant results are presented in Table 2 for mild exercise and in Table 3 for intense exercise.

**Figure 10:**
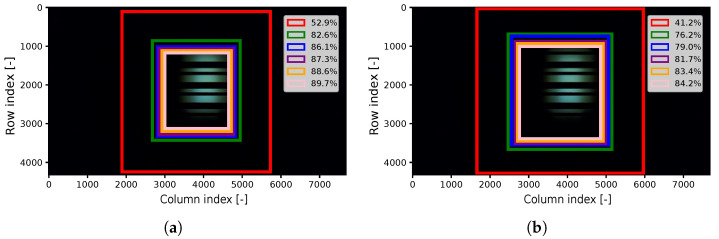
Random frame with the LED transmitter. The rectangles represent the percentage reduction of the scanning area in the frame. (**a**) Mild exercise. (**b**) Intense exercise.

The first columns of the tables display the percentage of position data considered along with their corresponding radius in pixels in one frame, as illustrated in Figure 9. Subsequently, the third and fourth columns present the percentage of data included within the radius of the LED $T_{x}$, as well as the percentage of data lost. Finally, the last column summarizes the percentage of reduction of the scanning area in the frame, depicted in Figure 10. In general, during intense exercise, the $T_{x}$’s wider range within the frame leads to an expansion of the scanning area.

It is evident that when all position-related data are considered, we do not lose any LED position in the frame, resulting in a reduction in the scanning area by 52.9% for mild exercise and 41.2% for intense exercise. On the contrary, when only 95% of the position data are considered, 35% and 38% of the data are lost for mild and intense exercise, respectively, despite the significant reduction in the scanning area, reaching 89.7% and 84.2%, respectively. By imposing a limitation on including 85% of the LED data to achieve a good accuracy in our system, we observe a reduction of 87.3% for mild exercise and 79.0% for intense exercise.

## 5. Conclusions

In this paper, we experimentally evaluate an OCC system utilizing a wearable LED transmitter. Evaluation is carried out under controlled user movement during physical exercise in an indoor setting. We demonstrate the feasibility of detecting the transmitting source within the frames. Finally, by analyzing the characteristics of controlled exercise-induced movement, we confine the tracking process to a smaller area within the image.

Our system is intended to oversee the activities of individuals, whether they are healthy or facing health issues, at sports training, elderly care, or rehabilitation. The obtained results highlight the significance of our system, as detecting the user’s position within the frame could offer valuable insights into their exercise intensity, age, gender, and uncover individual differences. Additionally, it has the potential to identify chronic conditions or detect early signs of injuries.

Although the proposed system has numerous advantages, there are various challenges that need further research to improve the effectiveness of the monitoring system. Primarily, there is a need to improve the hardware design of the wearable device to be light in weight, compact, user-friendly, waterproof and effortlessly incorporated into clothing or accessories, all without causing disruptions to user’s regular activities. Online video monitoring of individuals or multiple users in care units, gyms, or homes presents an additional challenge. However, it could offer people a sense of safety while engaging in their daily activities, knowing that they are being supervised in real time. Considerations for eye sensitivity with regard to light intensity must also be taken into account, especially in healthcare environments.

Future research will explore the relationship of the user’s position within the frame with factors such as exercise intensity, age, or gender. This exploration will involve comprehensive data analysis to uncover potential correlations and implications for personalized health monitoring. Additionally, we will investigate the efficacy of different transmitter technologies, including LED strips and fiber optics, to determine their suitability and performance in various scenarios. Moreover, understanding the influence of user movement on data transmission and reception will be a central point, as it can significantly impact the system’s reliability and accuracy. Furthermore, we plan to extend our experimental setup to encompass longer distances, enabling the evaluation of the system’s performance and robustness across larger spatial domains. This expansion will open up new possibilities for remote monitoring applications, promoting advancements in healthcare and beyond.

## Figures and Tables

**Table 1:** Parameters of the system and their values.

Module	Parameter	Value
$T_{x}$	Light source	LED array
	Device dimensions	11 × 6.5 × 3.5 cm
	Power supply	5 V
	Microcontroller	Seeeduino XIAO
		(Shenzhen, China)
	Illuminance	105 lux
Modulation	Modulation time	0.4 ms
	Data packet size	6b/packet [110100]
$R_{x}$	Smartphone camera	Samsung Galaxy S23
		(Suwon, Republic of Korea)
	Image sensor	S5KGN3
	Exposure time	83 μs
	Frame rate	30 fps
	ISO	125
	Resolution	7680 × 4320 px
Channel	Link distance *d*	20–30 cm

**Table 2:** Mild exercise. Percentage of position data considered, their corresponding radius in pixels, data included and lost from the LED transmitter and the percentage of reduction of the scanning area in the frame.

Position Data	Radius [px]	Data Included	Data Lost	Reduction
100%	1609	100%	0%	52.9%
99%	832	97%	3%	82.6%
98%	706	92%	8%	86.1%
97%	656	85%	15%	87.3%
96%	606	82%	18%	88.6%
95%	556	65%	35%	89.7%

**Table 3:** Intense exercise. Percentage of position data considered, their corresponding radius in pixels, data included and lost from the LED transmitter and the percentage of reduction of the scanning area in the frame.

Position Data	Radius [px]	Data Included	Data Lost	Reduction
100%	1841	100%	0%	41.2%
99%	1036	98%	2%	76.2%
98%	950	86%	14%	79.0%
97%	864	74%	26%	81.7%
96%	807	64%	36%	83.4%
95%	778	62%	38%	84.2%

## Data Availability

The data presented in this study are available on request from the corresponding author.
